# Impact of Quitting Smoking at Diagnosis on Overall Survival in Lung Cancer Patients: A Comprehensive Meta-Analysis

**DOI:** 10.3390/cancers17223623

**Published:** 2025-11-11

**Authors:** Jong Min Lee, Hyo-Weon Suh, Hyeon-Jeong Lee, Miyoung Choi, Ji Soo Kim, Kiheon Lee, Sang-Heon Kim, Jang Won Sohn, Ho Joo Yoon, Yu-Jin Paek, Cheol Min Lee, Dong Won Park

**Affiliations:** 1 Division of Pulmonary and Critical Care Medicine, Department of Internal Medicine, Seoul St. Mary’s Hospital College of Medicine, Catholic University of Korea, Seoul 06591, Republic of Korea; eco_sirius@naver.com; 2 Clinical Evidence Research Team, Division of Healthcare Research, National Evidence-Based Healthcare Collaborating Agency, Seoul 04933, Republic of Korealeehj@neca.re.kr (H.-J.L.); mychoi@neca.re.kr (M.C.); 3 Department of Family Medicine, Seoul National University College of Medicine, Seoul 03080, Republic of Korea; 4 Department of Family Medicine, Seoul National University Bundang Hospital, Seongnam 13620, Republic of Korea; 5 Division of Pulmonary Medicine and Allergy, Department of Internal Medicine, Hanyang University College of Medicine, Seoul 04763, Republic of Korea; 6 Department of Family Medicine and Health Promotion Center, Hallym University Sacred Heart Hospital, Anyang 14068, Republic of Korea; 7 Department of Family Medicine, Seoul National University Hospital Healthcare System Gangnam Center, Seoul 06236, Republic of Korea

**Keywords:** lung cancer, smoking cessation, survival, systematic review, meta-analysis

## Abstract

Background: Smoking cessation has been associated with reduced lung cancer mortality. This study aimed to synthesize current evidence on the impact of quitting smoking at or around the time of diagnosis of lung cancer on survival, considering factors such as histological subtype, cancer stage, and cessation intervention. Methods: A systematic search was conducted in the Ovid-MEDLINE, Ovid-EMBASE, Cochrane Central Register of Controlled Trials, and KoreaMed databases up to September 2024. Randomized controlled trials and cohort studies enrolling adult current smokers with pathologically confirmed lung cancer and comparing smoking cessation at or around diagnosis with continued smoking, were included. The primary outcome was overall survival (minimum follow-up of 3 months). The included studies were critically appraised using the revised Risk of Bias for Nonrandomized Studies (RoBANS 2) tool and meta-analyzed. Results: A total of 25 cohort studies comprising 17,584 patients were reviewed. Quitting smoking at diagnosis was associated with a 26% reduction in mortality risk (adjusted HR [aHR] 0.74, 95% CI 0.68–0.81). In subgroup analyses, quitting smoking was associated with improved survival in both non-small cell lung cancer (aHR 0.73, 95% CI 0.64–0.83) and small cell lung cancer (aHR 0.61, 95% CI 0.51–0.72), with a more pronounced benefit among patients with early-stage disease (stage I–III or limited stage; aHR 0.64, 95% CI 0.56–0.74). Furthermore, active smoking cessation interventions were significantly associated with improved survival (aHR 0.55, 95% CI 0.35–0.88). Conclusions: The findings underscore the importance of encouraging smoking cessation at the time of lung cancer diagnosis as an integral part of patient management to improve survival outcomes.

## Simple Summary

Quitting smoking at or around the time of the lung cancer diagnosis is thought to substantially improve survival, yet evidence from diverse studies has been inconsistent. This systemic review was conducted to clarify the survival benefit of quitting smoking at diagnosis. We analyzed 25 studies including 17,584 patients and found that patients who stopped smoking at diagnosis had a 26% lower risk of death compared with those who continued smoking. The survival benefit was observed across both NSCLC and SCLC and was particularly pronounced in early-stage disease. Importantly, active smoking cessation interventions were associated with the greater survival improvement. These findings highlight the critical role of integrating structured smoking cessation programs into lung cancer management and may guide future clinical practice and policy development.

## 1. Introduction

Lung cancer is among the most prevalent and lethal malignancies worldwide, accounting for a substantial proportion of cancer-related deaths in many countries. The economic burden of lung cancer prevention and treatment places a considerable strain on public health systems [1,2]. Among the various established risk factors, cigarette smoking is widely recognized as one of the most critical and modifiable etiological contributors to lung cancer [3]. Smoking after lung cancer diagnosis has been linked to increased cancer recurrence, second primary malignancies, and higher mortality [4]. Furthermore, continued smoking may exacerbate treatment-related complications and negatively affect quality of life [5,6].

Lung cancer comprises two major histological subtypes—non-small cell lung cancer (NSCLC) and small cell lung cancer (SCLC)—both strongly linked to cigarette smoking. Smoking promotes tumorigenesis through mutagenic and inflammatory pathways, while cessation may mitigate these effects and improve treatment responsiveness.

Quitting smoking has been shown to significantly reduce the risk of both lung cancer incidence and mortality [7]. A recent retrospective study by Fares et al., involving 42,087 patients with NSCLC, demonstrated that quitting smoking for at least 1 year before diagnosis was associated with improved survival outcomes [8]. However, the survival benefit of quitting smoking at the time of diagnosis, or shortly after, remains less well-defined. Moreover, evaluation of the clinical impact of quitting smoking on overall survival is complicated by the variability in treatment strategies based on histological subtype and cancer stage. Nevertheless, sustained smoking abstinence after diagnosis is known to significantly reduce lung cancer mortality, highlighting the importance of encouraging smoking cessation in the management of lung cancer patients.

The period surrounding the diagnosis of lung cancer represents a critical window for smoking cessation interventions, as patients are usually more receptive to behavior changes at this time. Observational studies have demonstrated that quitting smoking at or shortly after diagnosis may improve survival among lung cancer patients [4,9,10,11,12]. Furthermore, a meta-analysis by Caini et al. confirmed the survival benefit of quitting smoking post-diagnosis [13]. These findings underscore the importance of effective smoking cessation interventions and support in the care of newly diagnosed cancer patients [14].

To date, only a limited number of reviews have examined the impact of quitting smoking at or around the time of diagnosis on the survival outcomes of lung cancer patients. Moreover, no meta-analysis has systematically evaluated the effectiveness of active smoking cessation interventions in improving clinical outcomes for smokers diagnosed with lung cancer. Therefore, this study aimed to assess the effect of quitting smoking at or around the time of the diagnosis on overall survival among patients with different lung cancer types (NSCLC and SCLC) and stages. In addition, we evaluated the efficacy of active smoking cessation interventions for improving clinical outcomes in this patient population.

## 2. Materials and Methods

The study protocol was prospectively registered in the PROSPERO database (CRD42024583431) and conducted in accordance with the 2020 PRISMA (Preferred Reporting Items for Systematic Reviews and Meta-Analyses) guidelines [15]. The PRISMA 2020 checklist for the study is available in the Supplement Materials as Table S1.

### 2.1. Eligibility Criteria

Studies were eligible for inclusion if they met the following criteria: (1) Participants: adult patients (aged ≥ 18 years) with lung cancer who had smoked prior to diagnosis. Studies involving patients with any histological subtype or stage of lung cancer were eligible; (2) Intervention: smoking cessation at or around diagnosis (i.e., quitting smoking at diagnosis, at some point thereafter, or up to 12 months before diagnosis); (3) Comparator: continued smoking; (4) Outcomes: long-term overall survival or mortality (defined as follow-up duration > 3 months); (5) Study design: randomized controlled trials (RCTs) and cohort studies.

Smoking status at the time of diagnosis had to be clearly reported. Smoking cessation was verified either through self-report or biochemically confirmed according to criteria defined by the individual studies. The time period of quitting smoking 12 months before the diagnosis was selected based on prior meta-analysis [13]. The studies that employed active cessation intervention were included, which referred to structured smoking-cessation support involving behavioral counseling and/or pharmacotherapies as specified in each study. The frequency and duration of counseling sessions varied across cohorts but generally included individualized behavioral support delivered at or shortly after diagnosis. We excluded conference abstracts, preprints, editorials, and correspondence. There were no restrictions based on setting, geographic region, publication date, or language.

### 2.2. Search Strategy

We systematically searched Ovid-MEDLINE, Ovid-Embase, Cochrane Library, and KoreaMed (Korean database) in September 2024. The search strategy included the following key terms: (smokers OR nicotine dependence) AND (lung cancer) AND (smoking cessation) AND (survival) AND (RCT OR cohort study). A detailed search strategy for Ovid-MEDLINE is presented in Table S2. Each database-specific strategy was pilot-tested prior to the full search.

### 2.3. Selection Process

Four reviewers (Lee HJ, Choi M, Kim JS, and Lee K) independently screened the titles and abstracts for potentially relevant studies in pairs. After the initial screening, each pair of reviewers independently assessed the full-text articles for eligibility. Disagreements were resolved through discussion and consensus.

### 2.4. Data Collection Process

Two reviewers (Lee JM and Suh HW) independently extracted relevant data using a standardized form (Excel; Microsoft Corp.). The following information was extracted from each study: (1) Study characteristics: first author, year of publication, study design, country, setting, follow-up duration, and sample size; (2) Patient characteristics: lung cancer type and stage, age, sex, year of diagnosis, and cancer treatment; (3) Smoking status: timing of smoking cessation (before, at, or after diagnosis), smoking cessation intervention, method of smoking cessation verification; (4) Outcomes: adjusted hazard ratio (aHR), unadjusted hazard ratio (uHR), median survival time (MST), and survival rates. For studies that did not report hazard ratios (HRs) but provided Kaplan–Meier (KM) curves [16,17,18,19], HR values were estimated from the KM data using a method from a previously published meta-analysis [13]. This approach, based on the method proposed by Parmar et al. [20], allowed for the inclusion of studies that would otherwise be excluded, ensuring comprehensive representation of the available evidence.

### 2.5. Assessment of Risk of Bias

The Cochrane Risk of Bias tool (RoB 2) was pre-defined for the assessment of RCTs [21]. However, as no RCTs were included in this review due to lack of eligible trials, cohort studies were assessed for risk of bias using the Risk of Bias for Nonrandomized Studies (RoBANS 2) tool [22], which was originally developed in Korea in 2013 [23]. The eight domains of RoBANS 2 included baseline imbalance, selection of participants, control for confounding, measurement of intervention/exposure, blinding of outcome assessors, validity of outcome measurement, incomplete outcome data, and selective outcome reporting. Two reviewers (Lee JM and Suh HW) independently performed this assessment, and disagreements were resolved through discussion and consensus.

### 2.6. Synthesis Methods

We pooled the aHR and uHR with the 95% confidence interval (CI) for mortality using a random-effects model, provided that at least two studies were comparable [24]. Heterogeneity was assessed using the I^2^ statistic, with values of <25% considered low, 25–75% moderate, and >75% high [25]. Subgroup analyses were performed to explore potential sources of heterogeneity based on the aHR. The uHR was not used due to lack of adjustment for confounders. The meta-analysis was conducted using Review Manager (RevMan), version 5.4.1 (Cochrane Collaboration). The MST and survival rates were summarized qualitatively. To assess whether the risk of bias might have affected the results, we performed a sensitivity analysis including only studies that were rated as low risk across all RoBANS 2 domains.

### 2.7. Assessment of Publication Bias

We evaluated potential publication bias using a funnel plot and Egger’s test. If Egger’s test indicated statistical significance, the trim-and-fill method was applied to further assess publication bias [26]. All statistical analyses were conducted using R, version 4.4.3 (R Foundation for Statistical Computing).

## 3. Results

### 3.1. Study Selection

**Figure 1:**
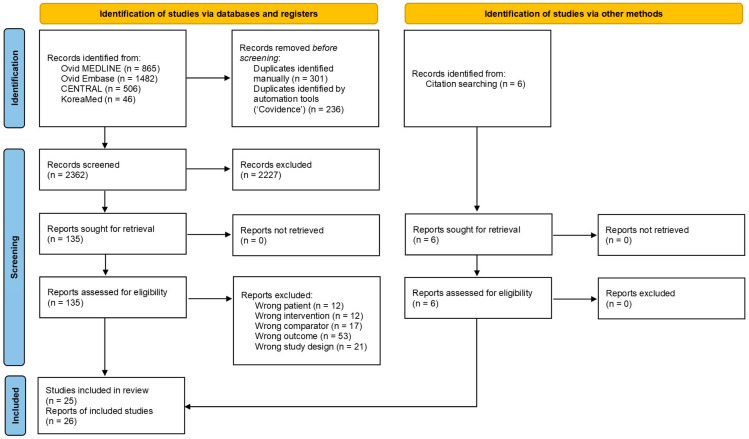
Flowchart of literature search and article selection for the systematic review and meta-analysis.

A total of 2899 records were identified from the databases, of which 537 duplicates were removed before screening. The titles and abstracts of 2362 records were screened, and 2227 records not relevant to this review were excluded. A total of 135 reports were identified through database searches, and an additional of 6 reports were found via citation searching. After reviewing the full-text, 115 reports were excluded. Ultimately, 25 cohort studies were included, corresponding to 26 reports. Two reports were from the same cohort study but with different observation periods [9,27]. The study selection process is illustrated in the PRISMA flow diagram (Figure 1).

### 3.2. Study Characteristics

The characteristics of the included studies are presented in Table S3. The review included 25 cohort studies from 12 countries across three continents. The majority of the studies originated from North America (48%, *n* = 12), followed by Europe (40%, *n* = 10) and Asia (12%, *n* = 3).

A total of 17,584 participants were included, with the proportion of male participants ranging from 40% to 96%. The mean age of participants ranged from 57 to 69 years. Participants received surgery, chemotherapy, radiotherapy, or no treatment at all, with the proportion of each treatment varying across the studies.

The majority of studies (52%, *n* = 13) enrolled patients with NSCLC, followed by studies involving both or unspecified subtypes (28%, *n* = 7) and SCLC (20%, *n* = 5). The proportion of patients with early-stage lung cancer ranged from 30% to 100%, except for 1 study including only patients with advanced-stage disease [19] and 3 studies that did not report staging information [11,28,29].

All studies compared mortality outcomes between smoking quitters and continued smokers. Biochemical verification of smoking cessation, defined as measuring urine cotinine levels or exhaled carbon monoxide (CO), was employed in 3 studies [19,27,30]. Smoking cessation interventions, including pharmacological or behavioral therapy, were incorporated in 6 studies [19,30,31,32,33,34]. The follow-up duration ranged from 1.75 to 27.7 years.

A total of 16 studies reported aHRs [4,11,12,19,27,28,29,31,32,33,35,36,37,38,39,40] and 7 reported uHRs [10,27,28,33,34,35,40], with 5 studies reporting both [27,28,33,35,40]. In 4 studies lacking HR data [16,17,18,19], uHR values were reconstructed from KM curves. Notably, a study [19] reported the aHR for the squamous cell carcinoma subgroup, whereas the uHR for the adenocarcinoma subgroup was estimated from KM data. The MST was reported in 12 studies [4,16,17,18,27,28,29,30,31,35,37,39] and survival rates were reported in 10 studies [4,10,27,32,33,34,35,36,39,41].

### 3.3. Risk of Bias in Included Studies

The risk of bias is summarized in Figures S1 and S2. Regarding baseline imbalance, over half of the studies (*n* = 13/25) were judged to have a high or unclear risk. Specifically, 6 studies reported baseline differences [27,29,32,37,38,42], and 7 studies did not report baseline characteristics for each group [10,11,12,31,40,41,43]. In terms of control for confounding, 32% of studies (*n* = 8/25) did not account for potential confounders (e.g., age, sex, and cancer stage) in their analyses [10,16,17,18,30,34,41,43]. For validity of outcome assessment, one study did not report how survival data were obtained [18]. In terms of selective outcome reporting, one study was judged to have an unclear risk, due to the lack of an accessible study protocol, with overall survival reported only by smoking cessation intervention (i.e., enrolled or declined) rather than smoking status (i.e., quit or continued); however, the MST for quitters and continued smokers was obtained directly from the authors [30]. All studies were deemed to have low risk of bias in the domains of selection of participants, measurement of intervention/exposure, blinding of outcome assessors, and incomplete outcome data.

Overall, concerns remained regarding baseline imbalance and control for confounding, which may limit the certainty of the pooled estimates.

### 3.4. Impact of Quitting Smoking at Diagnosis on Overall Survival

#### 3.4.1. Qualitative Analysis of Evidence

Among the 25 included studies, 64% of studies (*n* = 16/25) reported a survival benefit from quitting smoking at diagnosis. Of these studies, 14 studies demonstrated a statistically significant difference between quitters and continued smokers, primarily based on aHRs ranging from 0.34 to 0.85 [4,10,11,12,27,28,29,31,32,33,34,35,38,39]. The MST was reported in 7 studies, ranging from 1.5 to 6.6 years for quitters and from 1.0 to 4.8 years for continued smokers [4,27,28,29,31,35,39]. Unpublished data obtained from Park et al. [30] showed the following: NSCLC: 15, 52 ± 7.64 months for quitters vs. 4.97 ± 6.70 months for continued smokers; SCLC: 12.30 ± 6.13 months for quitters vs. 5.20 ± 5.49 months for continued smokers. Survival rates, reported in 8 studies, also favored quitters over continued smokers: 1-year survival, 70–88% vs. 54–82%; 2-year survival, 28–57% vs. 16–44%; 3-year survival, 34–96% vs. 25–81%; ≥5-year survival, 9–80% vs. 4–64% [4,10,27,32,33,34,35,39]. However, 8% of studies (*n* = 2/25) reported mixed results based on histological subtype or stage. A survival benefit was observed in squamous cell carcinoma and limited-stage SCLC but not in adenocarcinoma or extensive-stage SCLC [19,40]. The remaining 28% of studies (*n* = 7/25) showed no survival difference between quitters and continued smokers, likely due to the inclusion of patients with extensive-stage SCLC [17,18], multiple smoking categories [36,37], or inadequate adjustment for confounders [16,17,18,41,42]. The findings suggest that the survival benefit of quitting smoking at diagnosis may vary depending on subtype, stage, and confounding factors.

Subtype- and stage-specific analyses further support these observations. Among studies including both NSCLC and SCLC regardless of stage (*n* = 7), most of them reported a survival benefit from quitting smoking [10,11,28,29,30,31], with one exception [37]. For NSCLC of unspecified stage (*n* = 6), 4 studies demonstrated a benefit [12,27,32,34], and 2 studies did not show any benefit [16,36]. For early-stage NSCLC (stage I–III, *n* = 6), 4 studies found a benefit [33,38,39], and 2 studies did not observe any benefit [41,42]. A study on advanced-stage NSCLC (stage IV, *n* = 1) showed mixed results according to histological type, favoring squamous cell carcinoma [19]. For SCLC of unspecified stage (*n* = 3), 2 studies reported no difference between quitting and continued smoking [17,18], but one found benefit only in limited-stage patients [40]. In limited-stage SCLC (*n* = 2), both reported a survival benefit in quitters [4,35]. Taken together, the evidence suggests that quitting smoking at diagnosis may be associated with greater survival benefit in NSCLC, particularly in early-stage disease. In contrast, the evidence for survival benefit in SCLC is less conclusive, with improved survival primarily limited to limited-stage SCLC.

#### 3.4.2. Meta-Analysis

Of the 21 studies included in the meta-analysis, 16 studies reported the aHR [4,11,12,19,27,28,29,31,32,33,35,36,37,38,39,40]. The overall pooled estimate showed a 26% reduction in long-term all-cause mortality risk for quitters compared with continued smokers (aHR 0.74, 95% CI 0.67–0.81), as shown in Table 1 and Figure S3. On the other hand, the uHR was reported or estimated for 11 studies [10,16,17,18,19,27,28,33,34,35,40]. The overall pooled estimate showed a similar reduction in risk (uHR 0.75, 95% CI 0.66–0.84), as presented in Table 1 and Figure S4. Significant heterogeneity was observed in both analyses, as indicated by an I^2^ value greater than 50%.

#### 3.4.3. Subgroup Analyses

**Figure 2:**
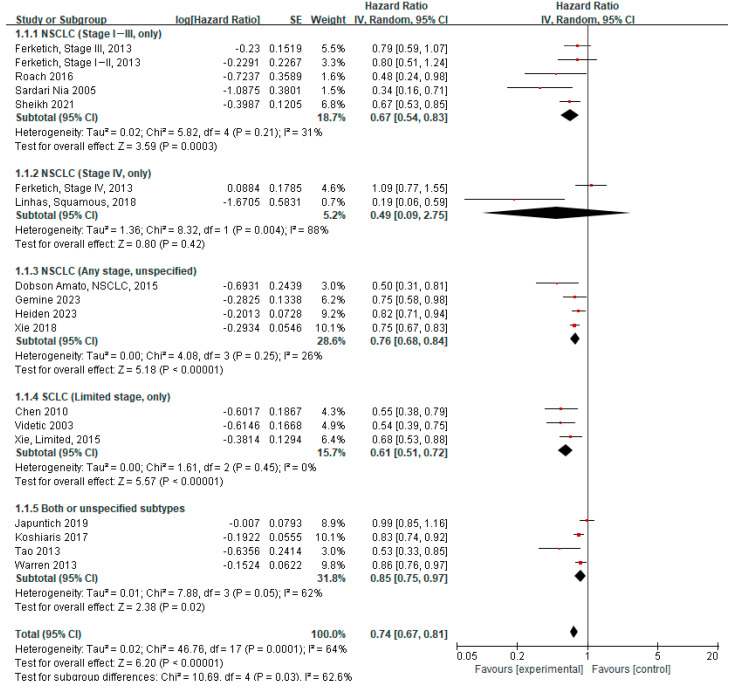
Forest plot of hazard ratios for quitters versus continued smokers at or around the time of diagnosis, stratified by cancer subtype and stage (adjusted). In this analysis, NSCLC subgroup data from Dobson Amato (2015) were included [4,9,11,12,19,28,29,31,32,33,35,36,37,38,39,40].

We conducted subgroup analyses with a focus on the aHR (Table 1 and Figure 2). We stratified studies by lung cancer subtype (NSCLC, SCLC, and both or unspecified). Furthermore, NSCLC was categorized into early stage (stages I–III) and advanced stage (stage IV), and SCLC was categorized into limited stage and extensive stage. For NSCLC, the overall pooled estimate showed a survival benefit associated with smoking cessation (aHR 0.73, 95% CI 0.64–0.83), which was more pronounced in early-stage NSCLC (aHR 0.67, 95% CI 0.54–0.83). However, no significant difference was observed between quitters and continued smokers in advanced-stage NSCLC. Given the wide confidence interval of the pooled estimate, additional studies are warranted before a definitive conclusion can be drawn (aHR 0.49, 95% CI 0.09–2.75). For SCLC, the survival benefit of quitting smoking was observed in the limited stage (aHR 0.61, 95% CI 0.51–0.72). Although no aHR data were available in the extensive stage, uHR data showed no significant difference between quitters and continued smokers (uHR 0.89, 95% CI 0.74–1.06). Overall, the survival benefit of quitting smoking was evident only in early-stage lung cancer but not in advanced-stage lung cancer (Table 1 and Figure S5).

**Figure 3:**
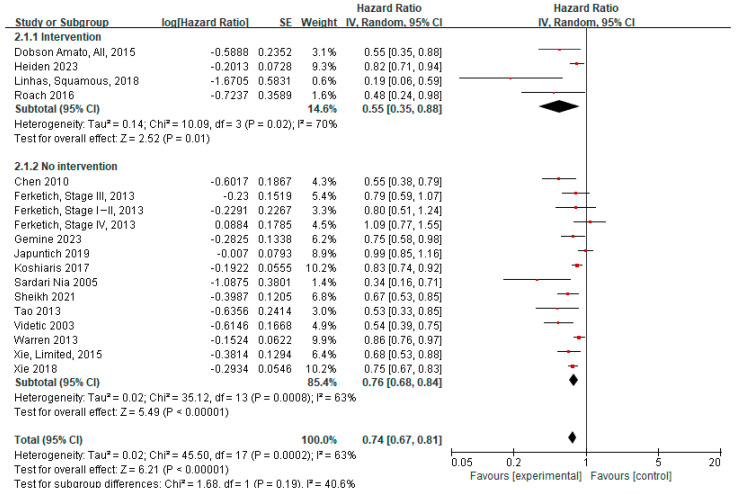
Forest plot of hazard ratios for quitters versus continued smokers at or around the time of diagnosis, stratified by smoking cessation intervention (adjusted). In this analysis, overall subject data rather than subgroup data from Dobson Amato (2015) were included [4,9,11,12,19,28,29,31,32,33,35,36,37,38,39,40].

In subgroup analyses stratified by smoking cessation intervention (intervention vs. no intervention; Figure 3), the survival benefit of quitting smoking was more pronounced in studies that incorporated smoking cessation interventions. The aHRs for mortality among quitters were 0.55 (95% CI 0.35–0.88) in studies with interventions and 0.76 (95% CI 0.68–0.84) in those without interventions.

**Figure 4:**
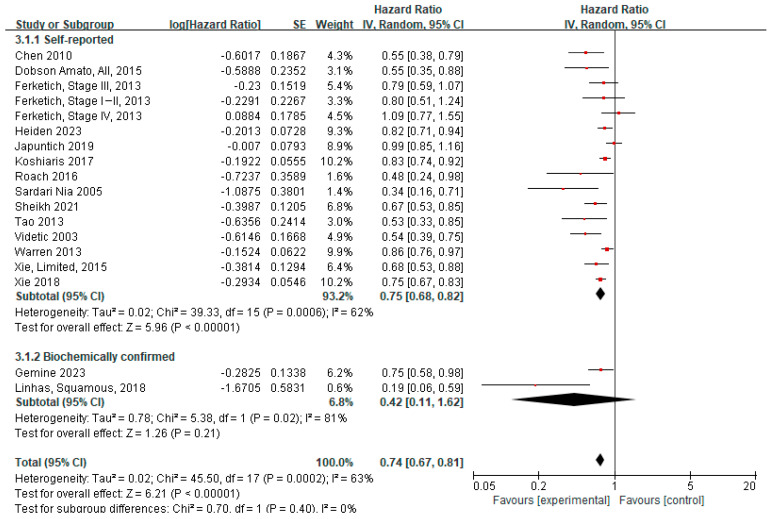
Forest plot of hazard ratios for quitters versus continued smokers at or around the time of diagnosis, stratified by method of smoking cessation verification (adjusted). In this analysis, overall subject data rather than subgroup data from Dobson Amato (2015) were included [4,9,11,12,19,28,29,31,32,33,35,36,37,38,39,40].

Furthermore, subgroup analyses stratified by method of smoking cessation verification (self-reported vs. biochemically confirmed; Figure 4) demonstrated the survival benefit of quitting smoking in studies with self-reported outcomes, with an aHR of 0.75 (95% CI 0.68–0.82). However, no significant difference in survival was observed between quitters and continued smokers in studies with biochemically confirmed outcomes (aHR 0.42, 95% CI 0.11–1.62).

#### 3.4.4. Sensitivity Analysis

To assess whether the risk of bias might have affected the results, we performed a sensitivity analysis including only studies that were rated as low risk across all RoBANS 2 domains. The pooled estimate remained consistent with the main analysis (aHR 0.70, 95% CI 0.59–0.84), indicating that the exclusion of studies at higher risk of bias did not materially change the overall results (Figure S6).

### 3.5. Publication Bias

The funnel plot and Egger’s test (*p* = 0.0068) indicated potential publication bias. Using the trim-and-fill method, 6 potentially missing studies were imputed, increasing the total number of studies from 16 to 22. After adjustment, the pooled aHR was increased slightly from 0.74 to 0.80; nevertheless, the association remained statistically significant (aHR 0.80, 95% CI 0.68–0.93). These findings suggest that minor publication bias may be present but did not materially affect the overall conclusion.

## 4. Discussion

In this systematic review of 25 cohort studies, quitting smoking was consistently associated with significant improvements in overall survival among lung cancer patients. Our meta-analysis, which included both adjusted and unadjusted HRs from 21 studies, further confirmed the survival advantage of quitting smoking. In subgroup analysis stratified by histological subtype, both NSCLC and SCLC patients gained significant survival advantages from smoking cessation. When stratified by clinical stage, a greater survival benefit was observed among patients with early-stage disease (stage I–III for NSCLC and limited stage for SCLC) compared with those with advanced-stage disease (stage IV for NSCLC and extensive stage for SCLC). Furthermore, studies incorporating active smoking cessation interventions consistently reported significantly better survival outcomes, highlighting the potential effectiveness of structured cessation support.

Unlike previous meta-analyses that evaluated smoking cessation primarily before diagnosis or without differentiating between clinical stages, our study provides novel and up-to-date insights by comprehensively assessing the survival impact of quitting smoking specifically at or around the time of lung cancer diagnosis, with additional subgroup analyses by histological subtype, clinical stage, and presence of active cessation interventions. This multidimensional approach provides novel insights into the optimal timing and clinical integration of smoking cessation in lung cancer care.

The results of our review align with the findings from previous studies. A systematic review by Parsons et al. analyzed 4 NSCLC and 2 SCLC observational studies and found that continued smoking post-diagnosis was associated with poorer disease-free and overall survival [44]. Similarly, a meta-analysis by Caini et al. of 8 NSCLC, 4 SCLC, and 6 mixed or unspecified subtype studies confirmed a significant survival benefit when smoking cessation occurred near the time of diagnosis [13]. Building upon these findings, our study further evaluated survival outcomes according to both histological subtype and clinical stage (I–III vs. IV in NSCLC; limited vs. extensive in SCLC) to identify subgroups most likely to benefit from quitting smoking at diagnosis. Notably, our findings demonstrated that the survival benefit of quitting was particularly pronounced in early-stage lung cancer, and no significant benefit was observed in advanced-stage lung cancer.

Quitting smoking at or around the cancer diagnosis may improve lung cancer survival through several interrelated biological mechanisms. Smoking has been shown to promote tumor progression, reduce treatment efficacy, and increase the risk of recurrence following therapy [45,46,47,48]. In addition to its oncogenic effects, smoking can contribute to various cardiorespiratory complications [49,50], providing a plausible biological rationale for the observed benefits of quitting smoking. The present analysis further suggests that the benefit of smoking cessation may be more pronounced in patients with early-stage lung cancer. This observation could be explained by several factors. Continued smoking after diagnosis increases the risk of postoperative complications and impairs wound healing, making cessation particularly advantageous in surgically treatable cases [51]. Furthermore, patients with early-stage disease may have greater motivation and adherence to cessation interventions, given the potential for curative treatment outcomes. Finally, at the molecular level, smoking induces epigenic alterations and increases tumor mutational burden in lung tissue [52,53]. Quitting smoking at an earlier stages of lung cancer, therefore, may allow partial reversal of smoking-related genetic and epigenetic alterations, thereby contributing to improved clinical outcomes [54].

A notable strength of our study is the evaluation of active smoking cessation interventions, an area previously unexplored in meta-analyses. Among the 25 studies included in this systematic review, 4 studies employed structured cessation programs and reported adjusted survival outcomes. In contrast to studies without cessation interventions, all 4 studies showed significantly better clinical outcomes, underscoring the clinical value of such programs. Quitting smoking is inherently difficult and often unsuccessful without appropriate support. Although most lung cancer patients attempt to quit after diagnosis [55], sustained abstinence is achieved by only a minority of patients [14,56]. A previous study by Warrant et al. reported that despite the acknowledgement of the clinical importance of smoking cessation in lung cancer care by most healthcare providers, only 39% of them provide active interventions [57]. Given the high relapse rate, timely support at the point of diagnosis is essential for improving cessation success and achieving favorable outcomes [14].

Of the 25 included studies, 4 studies were excluded from the meta-analysis due to insufficient or indirect survival data. For example, Lugg et al. [42] divided NSCLC patients into 4 groups (current smoker, ex-smoker < 6 weeks, ex-smoker ≥ 6 weeks, and never smoker) and reported no significant difference in postoperative survival. Rades et al. [41] examined the 1-year and 2-year overall survival rates of patients with early-stage NSCLC treated with radiotherapy but without adjustment for confounders, and they found no significant difference between quitters and continued smokers. Saito-Nakaya et al. [43] analyzed Japanese NSCLC patients and reported aHR of each continued smokers and quitters using never-smokers as the reference group, indirectly suggesting that quitting within 1 year of lung cancer diagnosis could improve survival. Park et al. [30] examined the effect of a smoking cessation program implemented during hospitalization for lung cancer treatment in South Korea by comparing the MST (months) of continued smokers and quitters. They found that the MST of quitters was significantly longer than that of continued smokers for both NSCLC and SCLC.

Although our study represents the most comprehensive systematic review on this topic to date, only 3 of the 25 included studies were conducted in Asian countries—one each from China, Japan, and South Korea [29,30,43]. The Western predominance may limit the generalizability of our findings. Furthermore, 2 of the 3 Asian studies were excluded from the meta-analysis due to their indirect methods of comparing the survival benefit between quitters and continued smokers. Nevertheless, both studies provided compelling evidence supporting the benefit of quitting smoking at or around the time of lung cancer diagnosis. Notably, the study by Tao et al. [29], the only Asian study included in the meta-analysis, demonstrated a significant survival benefit among quitters compared with continued smokers, consistent with the overall findings of our systematic review. These observations highlight the need for further well-designed studies in Asian populations to clarify the impact of smoking cessation at the time of lung cancer diagnosis in this region.

There are some limitations in our review. First, the inherent risk of bias and residual confounding in individual studies may have influenced the meta-analysis results. To mitigate the heterogeneity among included studies, we analyzed aHRs and uHRs separately, with both analyses consistently demonstrating favorable survival outcomes among quitters compared with continued smokers, supporting the robustness of our conclusion. However, because the adjustment variables for the reported hazard ratios varied across studies, residual confounding could not be entirely excluded despite adjustment for major prognostic factors in most cohorts. Second, most studies reported all-cause mortality instead of cancer-specific mortality, limiting our ability to determine whether the survival advantage was primarily driven by reduced cancer progression or by improvements in comorbid conditions. Future studies should aim to differentiate between cancer-related deaths and those due to cardiorespiratory or other causes to better evaluate the specific impact of cessation on lung cancer outcome. Third, the methods used to determine smoking cessation were not standardized. In most studies, cessation was confirmed through self-reports or retrospective chart reviews, and only 3 studies used biochemical verification with 2 of them providing aHRs. As shown in Figure 4, subgroup analysis of biochemically verified quitters resulted in a pooled estimate that failed to reach statistical significance, likely due to the small sample size, despite individual studies showing positive trends. Moreover, several studies assessed smoking status only once without follow-up, potentially introducing misclassification bias. Nevertheless, if smoking cessation truly improves survival, any misclassification—such as smokers being incorrectly categorized as quitters or quitters as smokers—would likely result in underestimation of actual survival benefit than our estimate. Although most included studies relied on self-reported smoking status, introducing some uncertainty, such misclassification would likely bias the pooled estimate toward the null. Therefore, our conclusion that smoking cessation is associated with improved survival remains consistent. Lastly, the timing of smoking cessation varied among the included studies, as the term “at diagnosis” encompassed different periods before and after diagnosis. Standardizing the cessation timeframe in future studies would help determine when smoking interventions are most effective.

## 5. Conclusions

Despite advances in chemotherapy and other therapeutic modalities for lung cancer, prevention and supportive care remain critical areas for research. Smoking cessation is a cornerstone of lung cancer management, and our study demonstrated its significant survival benefit, especially in early-stage disease. Importantly, our findings underscore the clinical importance of integrating active smoking cessation programs into routine clinical care, particularly at the time of diagnosis when patients may be more receptive to behavioral interventions. Future research should further evaluate the impact of smoking cessation on cancer-specific mortality in underrepresented populations, ideally using rigorous methods to adjust for confounders and collect detailed cause-of-death data. Given the high burden of smoking-related mortality, promoting and supporting cessation at the time of diagnosis should be a priority in comprehensive lung care management.

## Figures and Tables

**Table 1:** Pooled hazard ratios for quitters versus continued smokers at or around the time of diagnosis, stratified by cancer subtype and stage.

Lung Cancer Subtype	Subgroups/Total	Unadjusted HR	95% CI	*p* Value for Difference	Adjusted HR	95% CI	*p* Value for Difference
NSCLC	Stage I–III, only	0.42	0.25–0.72	0.002	0.67	0.54–0.83	0.0003
	Stage IV, only	1.17	0.77–1.77	0.47	0.49	0.09–2.75	0.42
	Any stage, unspecified *	0.75	0.55–1.01	0.06	0.76	0.68–0.84	<0.00001
	Overall pooled estimate	0.72	0.53–0.99	0.04	0.73	0.64–0.83	<0.00001
SCLC	Limited stage, only	0.53	0.37–0.77	0.0008	0.61	0.51–0.72	<0.00001
	Extensive stage, only	0.89	0.74–1.06	0.19	-	–	-
	Any stage, unspecified *	0.82	0.66–1.01	0.07	-	–	-
	Overall pooled estimate	0.78	0.63–0.95	0.01	0.61	0.51–0.72	<0.00001
Both or unspecified subtypes #	Overall pooled estimate	0.71	0.64–0.78	<0.00001	0.85	0.75–0.97	0.02
All studies †	Stage I–III or limited stage	0.50	0.37–0.67	<0.0001	0.64	0.56–0.74	<0.00001
	Stage IV or extensive stage	0.95	0.75–1.21	0.68	0.49	0.09–2.75	0.42
	Any stage, unspecified *	0.73	0.67–0.79	<0.00001	0.81	0.74–0.88	<0.00001
	Overall pooled estimate	0.75	0.66–0.84	<0.00001	0.74	0.67–0.81	<0.00001

* The included studies in “Any stage, unspecified” enrolled patients with lung cancer regardless of stage and they did not report the results separately according to the stage. # The included studies in “Both subtypes or subtype not specified” enrolled lung cancer regardless of cancer subtype and stage and they did not report the results separately according to the subtype or stage. † This included all studies on lung cancer, including NSCLC, SCLC, and both or unspecified subtypes.

## Data Availability

Data used in this study are available from the corresponding authors upon reasonable request.

## Supplementary Materials

The following supporting information can be downloaded at: https://www.mdpi.com/article/10.3390/cancers17223623/s1; Table S1: PRISMA 2020 checklist; Table S2: Search strategy for Ovid MEDLINE; Table S3: Main characteristics of the included studies; Figure S1: Risk of bias graph: review authors’ judgements about each risk of bias item presented as percentages across all included studies; Figure S2: Risk of bias summary: review authors’ judgements about each ‘Risk of bias’ item for each included study; Figure S3: Forest plot of hazard ratios for quitters versus continued smokers at diagnosis, stratified by cancer subtype (adjusted); Figure S4: Forest plot of hazard ratios for quitters versus continued smokers at diagnosis, stratified by cancer subtype (unadjusted); Figure S5: Forest plot of hazard ratios for quitters versus continued smokers at diagnosis, stratified by stage (adjusted); Figure S6: Sensitivity analysis including only studies with low risk of bias across all RoBANS 2 domains (adjusted).

## Abbreviations

The following abbreviations are used in this manuscript:

CI	Confidence interval
CO	Carbon monoxide
HR	Hazard ratio
KM	Kaplan–Meier
MST	Median survival time
NSCLC	Non-small cell lung cancer
PRISMA	Preferred reporting items for systematic reviews and meta-analyses
RCT	Randomized controlled trials
RevMan	Review Manager
RoB 2	Risk of bias tool 2
RoBANS 2	Risk of Bias for Nonrandomized Studies 2
SCLC	Small cell lung cancer
