# Supplementary material for: Impact of Quitting Smoking at Diagnosis on Overall Survival in Lung Cancer Patients: A Comprehensive Meta-Analysis
Source: Cancers (Basel). 2025 Nov 11;17(22):3623. doi: 10.3390/cancers17223623 (PMC12651382; doi:10.3390/cancers17223623)
Supplement: Supplementary file 1 [file cancers-17-03623-s001.zip › cancers-3962548-supplementary.pdf]

cancers

MDPI

Supplementary materials

Contents

Table S1…………………………………………………………………………………...………pg.2

Table S2…………………………………………………………………………………...………pg.4

Table S3…………………………………………………………………………………...………pg.5

Figure S1…………………………………………………………………………..……..…...……pg.8

Figure S2……………………………………………………………………...……....…...……pg.9

Figure S3…………………………………………………………………….....……………..……pg.10

Figure S4……………………………………………………………………...……..…......……pg.11

Figure S5……………………………………………………………………...……..…....…...…pg.12

Figure S6……………………………………………………………………...……..…....…...…pg.13

 https://doi.org/10.3390/cancers17223623

Table S1. PRISMA 2020 checklist.

Section and Topic	Item #	Checklist item	Location where item is reported
**TITLE**			
Title	1	Identify the report as a systematic review.	pg.1
**ABSTRACT**			
Abstract	2	See the PRISMA 2020 for Abstracts checklist.	pg.1-2
**INTRODUCTION**			
Rationale	3	Describe the rationale for the review in the context of existing knowledge.	pg.3
Objectives	4	Provide an explicit statement of the objective(s) or question(s) the review addresses.	pg.3
**METHODS**			
Eligibility criteria	5	Specify the inclusion and exclusion criteria for the review and how studies were grouped for the syntheses.	pg.3-4
Information sources	6	Specify all databases, registers, websites, organisations, reference lists and other sources searched or consulted to identify studies. Specify the date when each source was last searched or consulted.	pg.4
Search strategy	7	Present the full search strategies for all databases, registers and websites, including any filters and limits used.	pg.4
Selection process	8	Specify the methods used to decide whether a study met the inclusion criteria of the review, including how many reviewers screened each record and each report retrieved, whether they worked independently, and if applicable, details of automation tools used in the process.	pg.4
Data collection process	9	Specify the methods used to collect data from reports, including how many reviewers collected data from each report, whether they worked independently, any processes for obtaining or confirming data from study investigators, and if applicable, details of automation tools used in the process.	pg.4
Data items	10a	List and define all outcomes for which data were sought. Specify whether all results that were compatible with each outcome domain in each study were sought (e.g. for all measures, time points, analyses), and if not, the methods used to decide which results to collect.	pg.4
	10b	List and define all other variables for which data were sought (e.g. participant and intervention characteristics, funding sources). Describe any assumptions made about any missing or unclear information.	pg.4
Study risk of bias assessment	11	Specify the methods used to assess risk of bias in the included studies, including details of the tool(s) used, how many reviewers assessed each study and whether they worked independently, and if applicable, details of automation tools used in the process.	pg.4
Effect measures	12	Specify for each outcome the effect measure(s) (e.g. risk ratio, mean difference) used in the synthesis or presentation of results.	pg.4-5
Synthesis methods	13a	Describe the processes used to decide which studies were eligible for each synthesis (e.g. tabulating the study intervention characteristics and comparing against the planned groups for each synthesis (item #5)).	pg.4-5
	13b	Describe any methods required to prepare the data for presentation or synthesis, such as handling of missing summary statistics, or data conversions.	pg.4-5
	13c	Describe any methods used to tabulate or visually display results of individual studies and syntheses.	pg.4-5
	13d	Describe any methods used to synthesize results and provide a rationale for the choice(s). If meta-analysis was performed, describe the model(s), method(s) to identify the presence and extent of statistical heterogeneity, and software package(s) used.	pg.4-5
	13e	Describe any methods used to explore possible causes of heterogeneity among study results (e.g. subgroup analysis, meta-regression).	pg.4-5
	13f	Describe any sensitivity analyses conducted to assess robustness of the synthesized results.	pg.5
Reporting bias assessment	14	Describe any methods used to assess risk of bias due to missing results in a synthesis (arising from reporting biases).	pg.5

Section and Topic	Item #	Checklist item	Location where item is reported
Certainty assessment	15	Describe any methods used to assess certainty (or confidence) in the body of evidence for an outcome.	pg.4-5
**RESULTS**			
Study selection	16a	Describe the results of the search and selection process, from the number of records identified in the search to the number of studies included in the review, ideally using a flow diagram.	pg.5
	16b	Cite studies that might appear to meet the inclusion criteria, but which were excluded, and explain why they were excluded.	pg.5-6
Study characteristics	17	Cite each included study and present its characteristics.	pg.6
Risk of bias in studies	18	Present assessments of risk of bias for each included study.	pg.6
Results of individual studies	19	For all outcomes, present, for each study: (a) summary statistics for each group (where appropriate) and (b) an effect estimate and its precision (e.g. confidence/credible interval), ideally using structured tables or plots.	pg.6-7
Results of syntheses	20a	For each synthesis, briefly summarise the characteristics and risk of bias among contributing studies.	pg.6-7
	20b	Present results of all statistical syntheses conducted. If meta-analysis was done, present for each the summary estimate and its precision (e.g. confidence/credible interval) and measures of statistical heterogeneity. If comparing groups, describe the direction of the effect.	pg.7
	20c	Present results of all investigations of possible causes of heterogeneity among study results.	pg.7
	20d	Present results of all sensitivity analyses conducted to assess the robustness of the synthesized results.	pg.8
Reporting biases	21	Present assessments of risk of bias due to missing results (arising from reporting biases) for each synthesis assessed.	pg.10
Certainty of evidence	22	Present assessments of certainty (or confidence) in the body of evidence for each outcome assessed.	pg.6-7
**DISCUSSION**			
Discussion	23a	Provide a general interpretation of the results in the context of other evidence.	pg.10-11
	23b	Discuss any limitations of the evidence included in the review.	pg.11-12
	23c	Discuss any limitations of the review processes used.	pg.12
	23d	Discuss implications of the results for practice, policy, and future research.	pg.12
**OTHER INFORMATION**			
Registration and protocol	24a	Provide registration information for the review, including register name and registration number, or state that the review was not registered.	pg.3
	24b	Indicate where the review protocol can be accessed, or state that a protocol was not prepared.	pg.3
	24c	Describe and explain any amendments to information provided at registration or in the protocol.	-
Support	25	Describe sources of financial or non-financial support for the review, and the role of the funders or sponsors in the review.	pg.13
Competing interests	26	Declare any competing interests of review authors.	pg.13
Availability of data, code and other materials	27	Report which of the following are publicly available and where they can be found: template data collection forms; data extracted from included studies; data used for all analyses; analytic code; any other materials used in the review.	pg.13

Table S2. Search strategy for Ovid MEDLINE.

Category	Line	Terms	Hit
Patient (P)	1	exp "Tobacco Use"/ OR exp "Tobacco Use Disorder"/ OR exp Smokers/ OR exp Smoking/ or (Tobacco or Tobacco-use* or smoker* or smoking or cigar or cigarett*).tw,kw.	442456
	2	((nicotine or tabacco) adj2 (addiction* or abuse or dependenc* or disorder*) or nicotinism).tw,kw	9672
	3	exp Lung Neoplasms/ OR ((lung or pulmonary or bronchopulmonary) adj3 (cancer or tumor* or tumour* or adenocarcinoma* or carcinoma* malignan* neoplasm* or tumo* or non?small?cell* or NSCLC or SCLC or small?cell* or oat?cell*)).tw,kw	380940
	4	(1 OR 2) AND 3	31773
Intervention (I)	5	exp Smoking Cessation/ OR exp "Tobacco Use Cessation"/ OR (smoking cessation* OR (quit* adj smoking) OR (giving up smoking) OR (stop adj smoking) or (smoking AND (quit* or stop* or give?up or giving?up or cessation* or ceas* or cutt* or cut or abstinence or abstain*))).tw,kw	64159
P & I	6	4 AND 5	3236
Outcome (O)	7	survival/ OR exp Survival Analysis/ OR exp prognosis/ OR exp Disease Progression/ OR exp Mortality/ OR (surviv* OR prognos* OR progress* OR mortalit* OR death OR outcome*).tw,kw	7232196
P & I & O	8	6 AND 7	1882
RCT filter	9	(Randomized Controlled Trials as Topic/ or randomized controlled trial/ or Random Allocation/ or Double Blind Method/ or Single Blind Method/ or clinical trial/ or clinical trial, phase i.pt. or clinical trial, phase ii.pt. or clinical trial, phase iii.pt. or clinical trial, phase iv.pt. or controlled clinical trial.pt. or randomized controlled trial.pt. or multicenter study.pt. or clinical trial.pt. or exp Clinical Trials as topic/ or (clinical adj trial$).tw. or ((singl$ or doubl$ or treb$ or tripl$) adj (blind$3 or mask$3)).tw. or PLACEBOS/ or placebo$.tw. or randomly allocated.tw. or (allocated adj2 random$).tw.) not (case report.tw. or letter/ or historical article/)	1968978
Cohort study filter	10	Epidemiologic Studies/ or exp Case Control Studies/ or exp Cohort Studies/ or Case-control.tw. or (cohort adj (study or studies)).tw. or Cohort analy$.tw. or (Follow up adj (study or studies)).tw. or (observational adj (study or studies)).tw. or Longitudinal.tw. or Retrospective.tw.or Cross sectional.tw. or Cross-sectional studies/	4087599
Study design (SD) filter	11	9 OR 10	5530131
P & I & O & SD filter	12	8 AND 11	865

Table S3. Main characteristics of the included studies.

Author (year) country	Stage I-III or Limited stage (%)	Men (%)	Age,yr	Treatment	Total (n)	Quitters (n)	Continued smokers (n)	Methods to smoking cessation assessment	Smoking cessation intervention	follow-up (years, maximum)
NSCLC										
Baser (2006) USA	I-III (90.3%)	49.5%	mean 62.3	surgery (61.9%), nonsurgical treatment (38.1%)	93	46	47	smoking history, medical record based on patient self-reporting	none	5
Doerr (2024) Germany	I-III (100%)	65.0%	mean 63.7	surgery (100%)	90	60	30	patient reports one year after the operation	structured smoking cessation program including behavioral therapy and optional nicotine replacement therapy	8
Ferketich (2013) USA	I-III (73.2%)	52.1%	mean 61.2	surgery (32.8%), CHT (65.8%)	1863	1483	380	smoking history, medical record	none	5
Gemine (2023) UK	I-III (57.6%)	59.3%	median 67 (range 59-73)	curative intent surgery (12.7%), radical RT (12.9%)	646	147	499	self-reported smoking status, exhaled CO measurement	none	2
Heiden (2023) USA	I-III (98.5%)	96.0%	mean 66.12	surgery (100%)	6168	662	5506	Health Factors data (nationally collected automated clinical prompts)	cessation pharmacotherapy (21.9%)	5
Linhas (2018) Portugal	I-III (0%)	91.8%	mean 57.0	CHT (100%)	97	50	47	exhaled CO measurement	a brief intervention for smoking cessation and a specialized consultation	Squamous: 1.75 Adeno: 3.3
Lugg (2017) UK	I-III (100%)	62.6%	mean 68.8	surgery (100%)	166	55	111	patient self-reporting	none	3.3
Rades (2008) Germany	I-III (100%)	78.5%	47%, aged >65	surgery (28.7%), CHT (40.9%), RT (100%)	181	107	74	NR	none	5.3
Roach (2016) USA	I (100%)	41.0%	median 67 (range 51–85)	SBRT (100%)	119	32	87	smoking history, questionnaire	free counseling services or smoking cessation program, which included prescription recommendations, and an optional six-week program on behavior modification	7.25
Saito-Nakaya (2006) Japan	I-III (100%)	60.9%	mean 62.4	surgery (100%)	98	92	6	NR	none	7.6
Sarda-riNia (2005) Belgium	I-III (100%)	86.3%	median 63	surgery (100%)	204	35	169	two questionnaires by the patient and the medical doctor	none	11.25
Sheikh (2021) Russia	I-III (100%)	88.5%	mean 61.3	surgery (88.3%), CHT (22.4%), RT (21.8%)	517	220	297	patient interview	none	13

Xie (2018) USA	I-III (62%)	55.0%	median 68 (range 24–97)	surgery (36%), CHT (15%), RT (8%), CHT–RT (12%), surgery + CHT, RT, or both (7%), other (2%), none (21%)	1779	904	875	smoking history, medical record	none	17.2
SCLC										
Bergman (1988) Denmark	Limited (51.9%)	65.6%	mean 60	surgery (5.2%), CHT (100%)	122	51	71	smoking history, clinical evaluation	none	3.3
Chen (2010) USA	Limited (100%)	NR	mean 65.1	surgery with or without CHT, RT, or both (12.7%), CHT or RT (82.3%), none (1.1%)	163	87	76	smoking history, medical record	none	7.5
John-ston-Early (1980) USA	Limited (30.4%)	NR	NR	CHT with or without RT (% not given)	92	35	57	smoking history, obtained from pa-tients or family member	none	4
Videtic (2003) Canada	Limited (100%)	58.6%	median 63 (range 32–94)	CHT–RT (100%)	186	107	79	NR	none	7
Xie (2015) USA	Limited (40.8%)	53.3%	median 68 (range 27–91)	CHT or RT (39.1%), CHT–RT (40.5%), surgery + adju-vant therapy (6.2%), none (14.1%)	570	287	283	smoking history, medical record	none	5
Both or unspecified subtypes										
Dobson Amato (2015) USA	I-III (65.2%)	40.2%	mean 61.9	NR	224	95	129	smoking history, medical record	tobacco cessation coun-seling service, which provide behavioral counseling with linkage to pharmacologic sup-port.	3.3
Japun-tich (2019) USA	I-III (71.0%)	48.3%	24.4%, aged ≥70	surgery (46.8%), CHT (67.1%), RT (41.1%)	1067	711	356	surveys using computer assisted telephone inter-views	none	7
Koshi-aris (2017) UK	NR	49.8%	mean 66.8	surgery (23.1%), CHT (29.2%), RT (15.5%)	1913	784	1129	smoking history, medical record	none	15
Kovacs (2012) Hungary	I-III (61%)	56.0%	NR	NR	499	284	215	questionnaire	none	2.5
Tao (2013) China	NR	NR	mean 66.9	surgery, CHT, RT in various	184	NR	NR	patient interview	none	5.3

				combinations (% not given)						
Warren (2013) USA	NR	60.8%	mean 61.9	NR	470	249	221	questionnaire	none	27.7
Park (2020) Korea	I-III (69.9%)	91.8%	Mean 66.0	surgery (27.4%), CHT (57.5%), RT (50.7%)	73	39	34	urine cotinine and exhaled CO measurement	inpatient smoking cessation program+ regular counseling for 6months after discharge	2

In the study by Heiden et al., Corporate Data Warehouse pharmacy outpatient records were reviewed for prescriptions for smoking cessation pharmacotherapy, which revealed that 29.91% of quitters and 20.94% of continuing smokers had been prescribed such medication, but no active smoking cessation treatment was implemented in the study. The study by Saito-Nakaya et al. was the only one in which the survival of quitters and continued smokers was not compared directly, but instead used a third group of patients (never-smokers) as the reference group.

CHT, chemotherapy; CO, carbon monoxide; LC, lung cancer; NR, not reported; RT, radiotherapy; SBRT, stereotactic body radiation therapy.

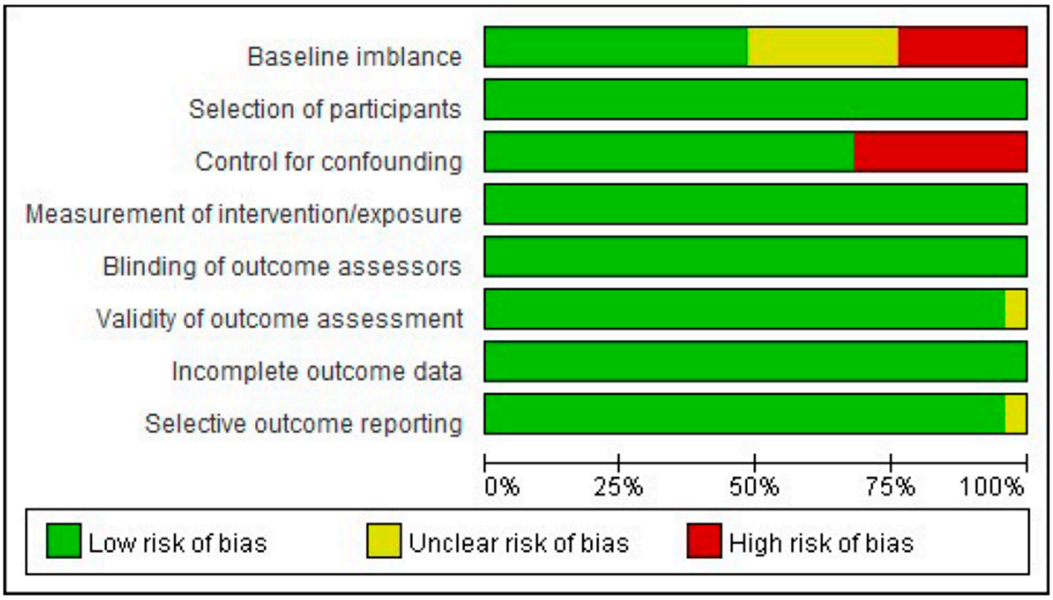

Figure S1. Risk of bias graph: review authors' judgements about each risk of bias item presented as percentages across all included studies.

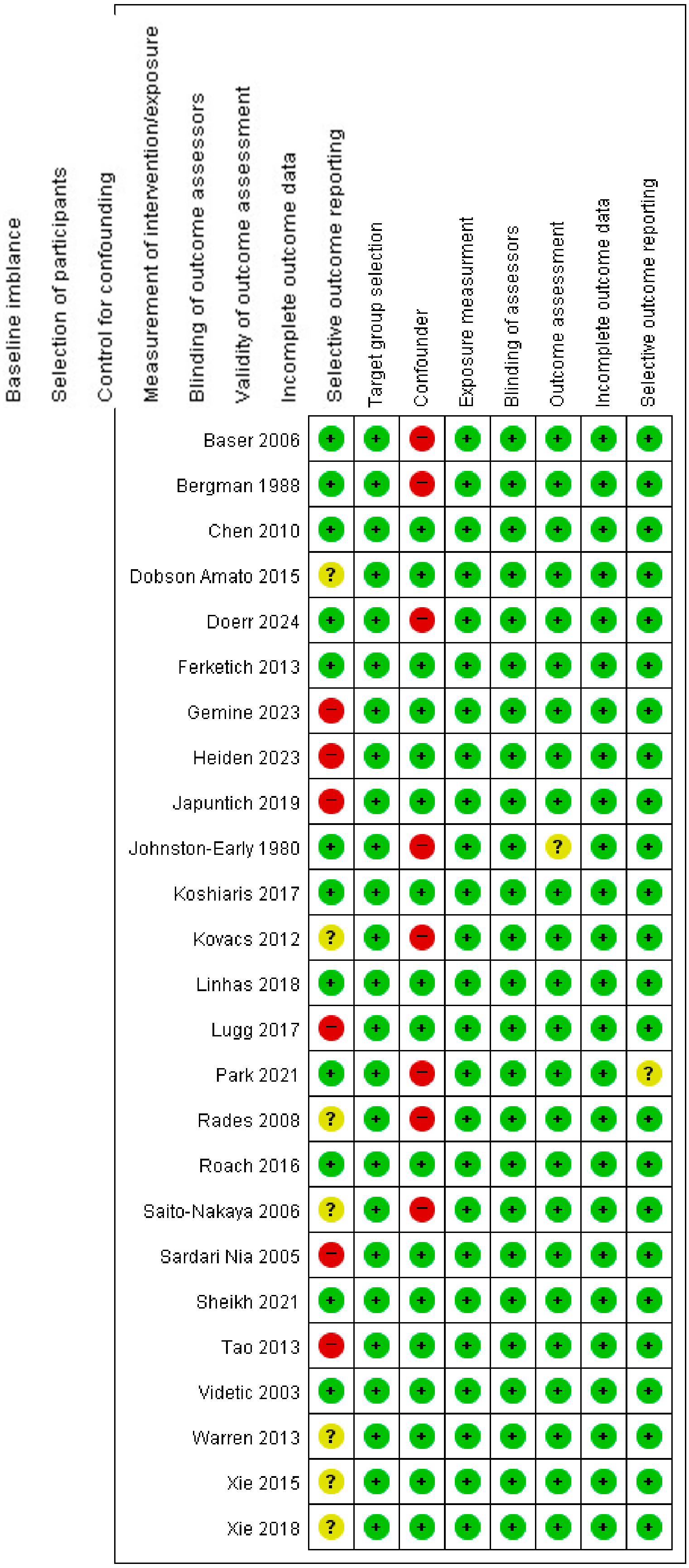

Figure S2. Risk of bias summary: review authors' judgements about each 'Risk of bias' item for each included study.

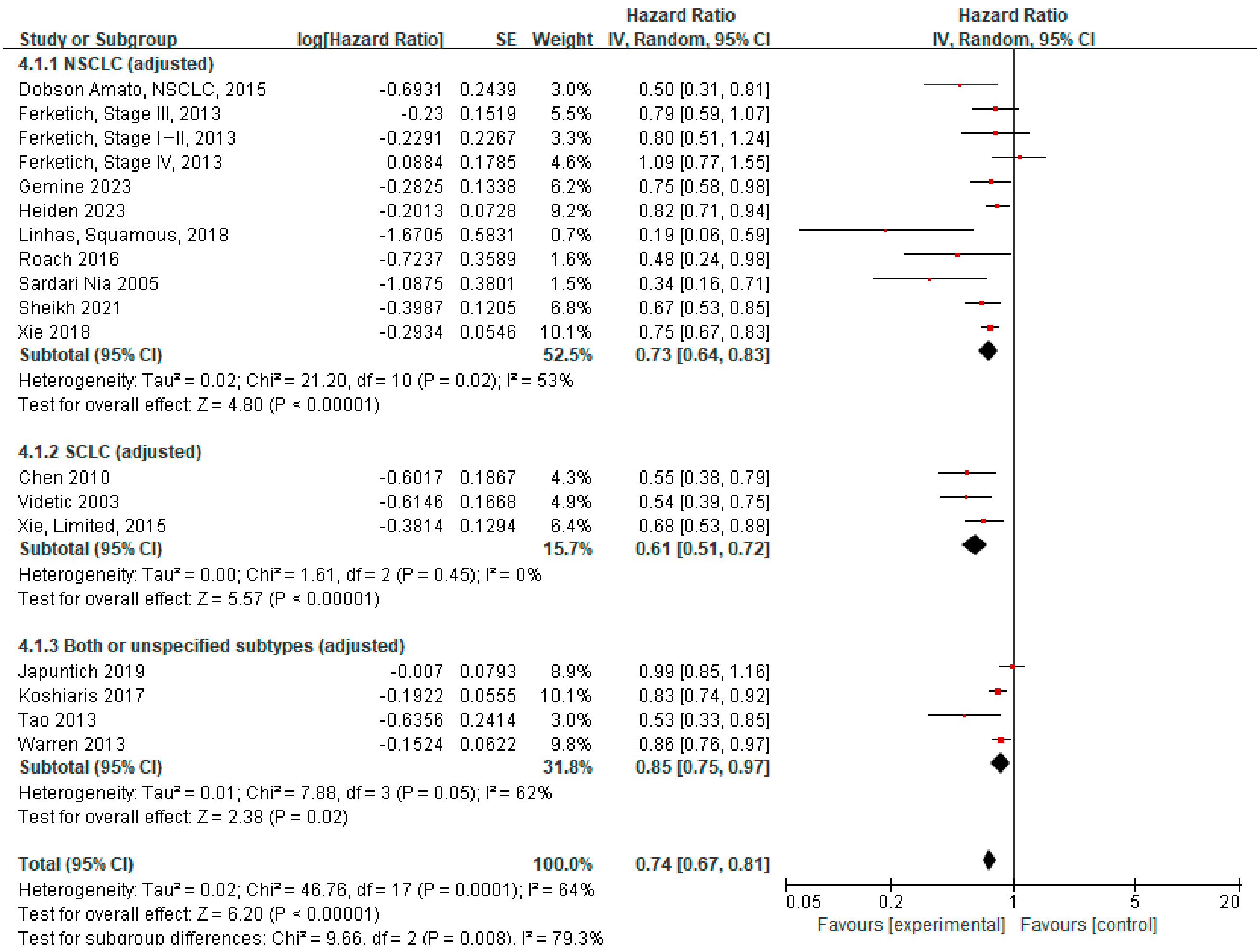

Figure S3. Forest plot of hazard ratios for quitters versus continued smokers at diagnosis, stratified by cancer subtype (adjusted). In this analysis, NSCLC subgroup data rather than overall subject data from Dobson Amato (2015) were included.

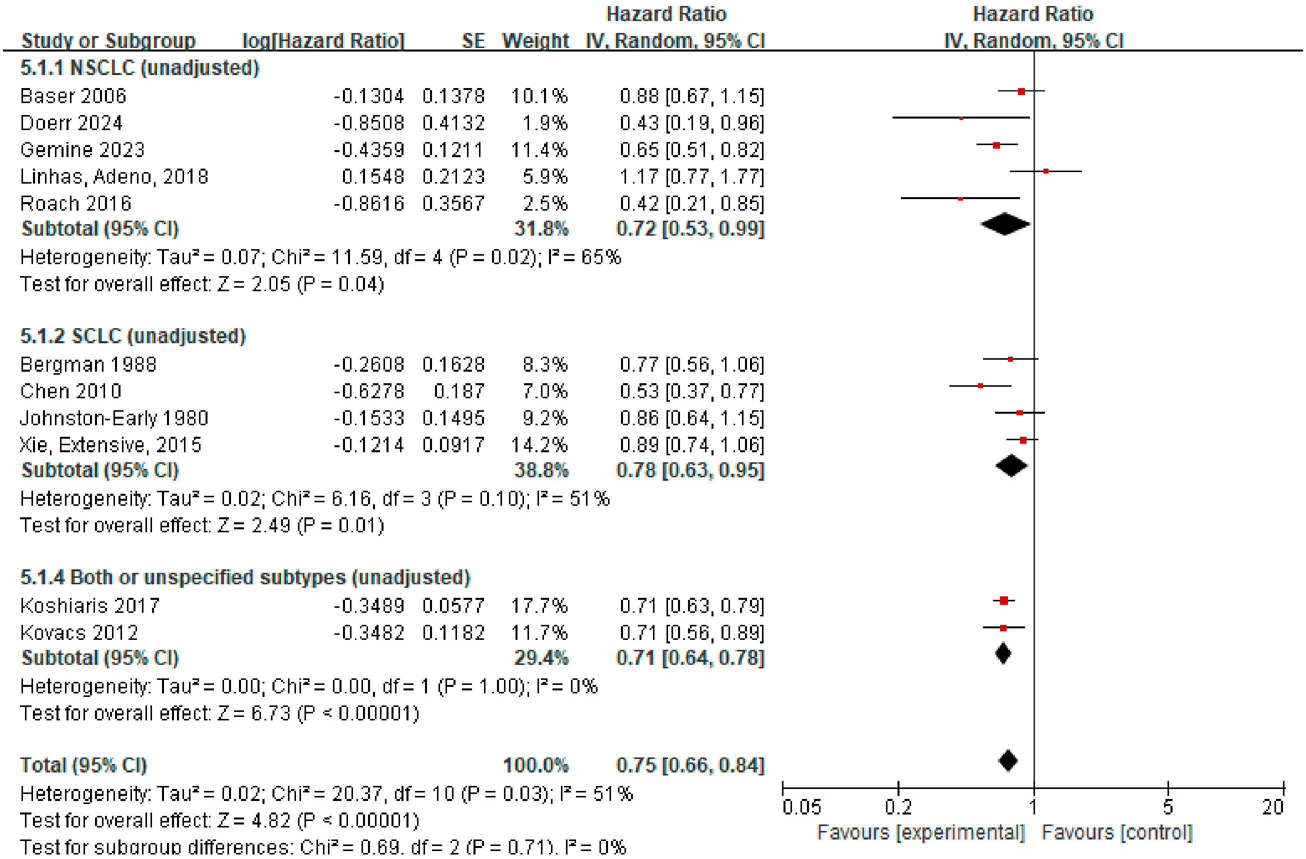

Figure S4. Forest plot of hazard ratios for quitters versus continued smokers at diagnosis, stratified by cancer subtype (unadjusted).

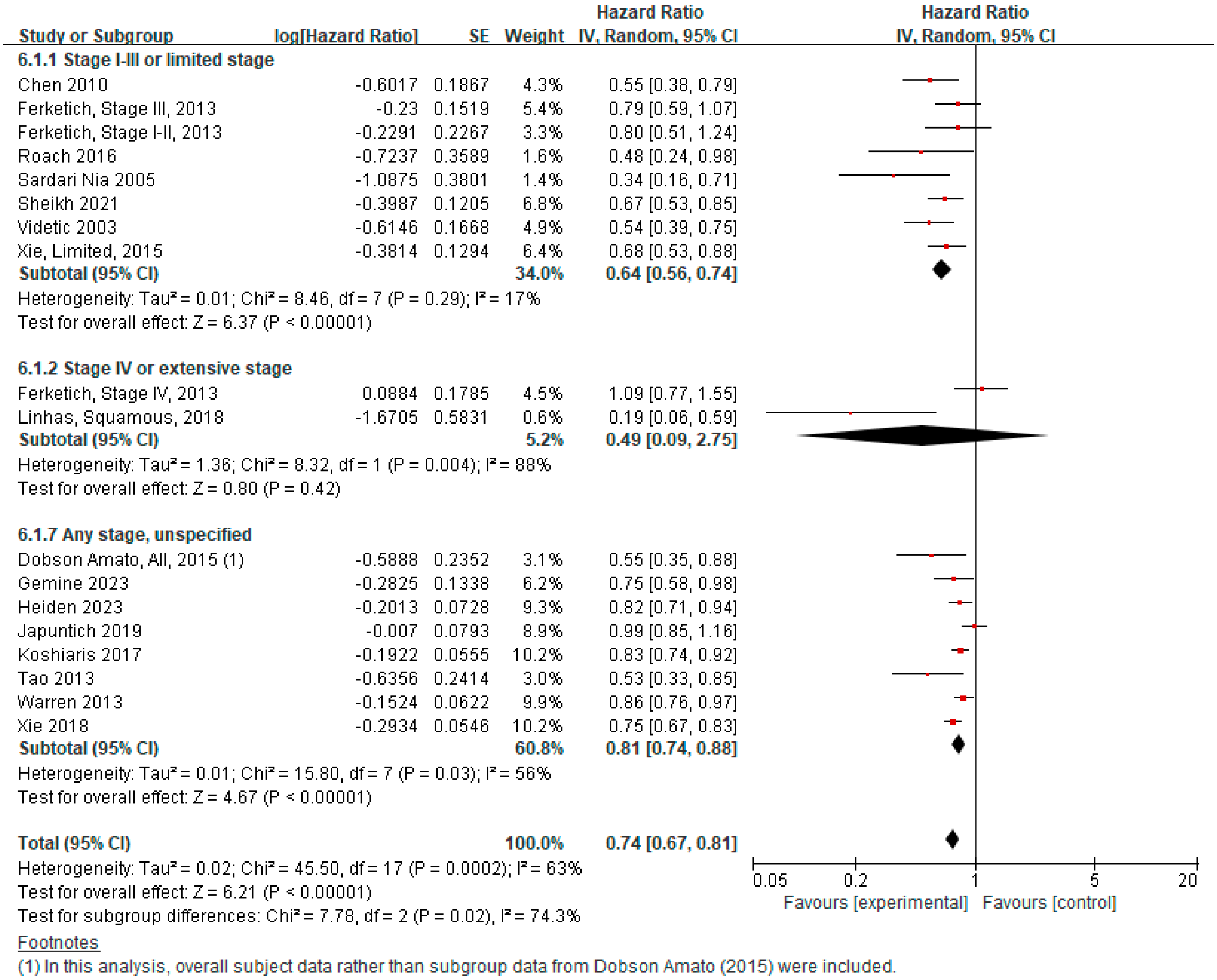

Figure S5. Forest plot of hazard ratios for quitters versus continued smokers at diagnosis, stratified by stage (adjusted). In this analysis, overall subject data rather than subgroup data from Dobson Amato (2015) were included.

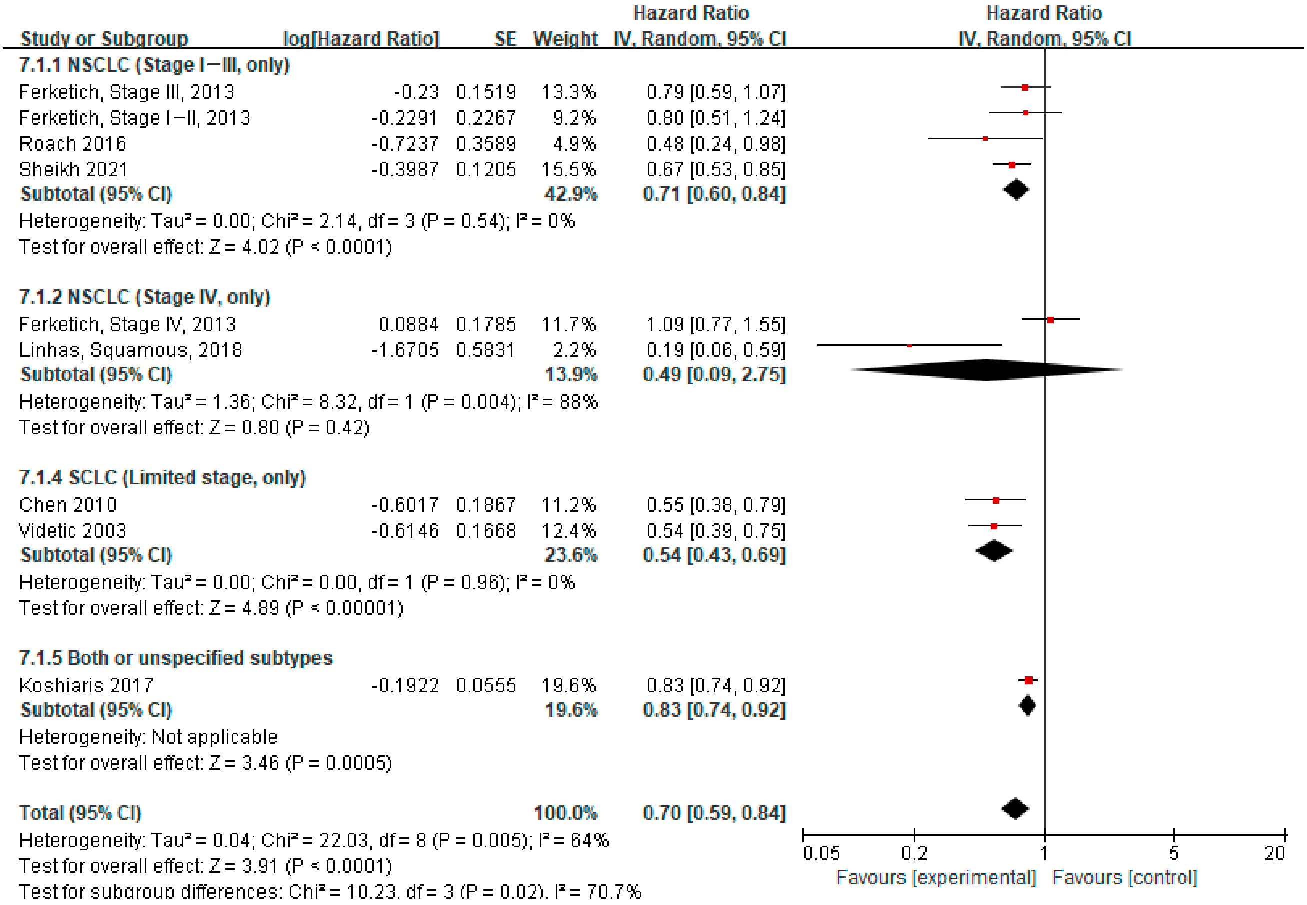

Figure S6. Sensitivity analysis including only studies with low risk of bias across all RoBANS 2 domains (adjusted).
